# Impact of fractional CO_2_ laser therapy on vaginal wall histology in breast cancer survivors

**DOI:** 10.1007/s10103-026-04829-0

**Published:** 2026-02-10

**Authors:** Sine Jacobsen, Mary Holten Bennetsen, Patricia Switten Nielsen, Marianne Glavind-Kristensen, Anders Bonde Jensen, Axel Forman, Marianne Waldstrøm, Pinar Bor

**Affiliations:** 1 Department of Obstetrics and Gynaecology, Regional Hospital Randers, Randers, Denmark; 2 Department of Clinical Medicine, Aarhus University Hospital, Aarhus, Denmark; 3 Department of Pathology, Aarhus University Hospital, Aarhus, Denmark; 4 Department of Obstetrics and Gynaecology, Aarhus University Hospital, Aarhus, Denmark; 5 Department of Oncology, Aarhus University Hospital, Aarhus, Denmark

**Keywords:** Fractional CO₂ laser therapy, Breast cancer survivors, Genitourinary syndrome of menopause, Histology, Collagen, Elastin

## Abstract

To investigate histological changes in the vaginal wall of breast cancer survivors (BCSs) with genitourinary syndrome of menopause (GSM) treated with five sessions of fractional CO₂ vaginal laser therapy, focusing on epithelial morphology and stromal extracellular matrix composition. In this prospective single-center study, 26 BCSs underwent five sessions of fractional CO_2_ vaginal laser therapy. Vaginal punch biopsies were collected at baseline and four to six weeks after completing the final session. Mucosal morphology was classified (Type 1, 2, or 3) independently by two pathologists, blinded to baseline and follow-up status. Stromal elastin, total collagen, and collagen types I and III were quantified by digital image analysis of Verhoeff–Van Gieson, Masson’s trichrome, and immunohistochemistry. Paired tests and the Stuart–Maxwell test evaluated changes over time. At baseline, 42.3% of participants were classified as Type 1, 19.2% as Type 2, and 38.5% as Type 3 mucosal morphology. Elastin index increased from 33.1% to 56.8% (*p* < 0.01), and collagen type III index from 44.5% to 58.4% (*p* = 0.03), while total collagen and collagen type I showed no significant change. No significant differences in epithelial microscopic features were observed (Stuart-Maxwell *p* = 0.16). Fractional vaginal CO₂ laser therapy was associated with selective stromal remodelling in BCSs - higher elastin and collagen type III indices, but without detectable improvement in epithelial morphology. Given the absence of a control group, the short follow-up, and limited epithelial morphometry due to section orientation, these findings should be interpreted as preliminary histological evidence of a stromal response rather than a confirmation of functional or regenerative benefit.

**Registry**: ClinicalTrials.gov, TRN: (NCT06007027). Registration date: 1 August 2023.

**Supplementary Information:**

The online version contains supplementary material available at 10.1007/s10103-026-04829-0.

## Introduction

Genitourinary syndrome of menopause (GSM) is characterised by symptoms including vaginal dryness, burning, recurrent urinary tract infections, and dyspareunia [1]. These symptoms result from oestrogen deficiency, which causes thinning of the vaginal epithelium, reduced vascularity, and decreased collagen and elastin content in the connective tissue [2]. Pelvic examination typically reveals a pale, dry vaginal epithelium with a smooth, shiny surface and an absence of tissue rugae. Up to 75% of breast cancer survivors (BCSs), particularly those receiving endocrine therapy, experience GSM-related symptoms [3]. Oestrogen-containing treatments are often problematic, as they may compromise the efficacy of endocrine therapy and increase the risk of recurrence, leaving a therapeutic gap [4].

Over the past decade, fractional CO_2_ laser therapy has emerged as a potential non-hormonal intervention for alleviating GSM symptoms. The treatment delivers micro-ablative pulses of energy to the vaginal wall, creating controlled zones of thermal injury that stimulate a wound-healing response [5–7]. Several studies in postmenopausal women have reported symptomatic improvement, but knowledge about the underlying mechanisms and histological effects on the vaginal wall remains limited. While the U.S. FDA in 2018 cautioned against unregulated marketing of ‘vaginal rejuvenation’ lasers due to limited evidence and reports of adverse events, such statements primarily addressed cosmetic use outside clinical research settings. The present study was conducted under full ethical approval.

Most prior work on laser therapy has focused on patient reported symptoms rather than tissue-level effects, despite the importance of histology for understanding mechanisms and long-term safety assessment [2, 7–10]. Histological findings have been inconsistent, small, uncontrolled series describing epithelial thickening and increased collagen or elastin after CO_2_ laser therapy [7–10], whereas more recent randomized controlled trials - such as those by Mension et al. [11] and Cantarelli et al. [12] – found no significant differences between active and sham or comparator treatments in BCSs. Notably, several studies also report a discrepancy between GSM symptom burden and histological appearance: even among highly symptomatic women, the vaginal epithelium may appear well estrogenised [12, 13]. This suggests that GSM in BCSs may reflect multifactorial or yet poorly understood mechanisms beyond epithelial thinning alone.

The aim of this study was to investigate histological changes in the vaginal wall of BCSs after five sessions of vaginal CO₂ laser therapy. Specifically, we examined epithelial morphology and extracellular matrix composition (elastin, total collagen, and collagen type I and III) using digital image analysis. Rather than evaluating clinical outcomes, this exploratory histological analysis was designed to describe epithelial and stromal responses and to inform the design of future controlled trials integrating clinical endpoints.

## Methods

### Study design and setting

This was a single-center, prospective exploratory histology study conducted at the Department of Gynaecology and Obstetrics, Randers Regional Hospital, Denmark (February 2023-April 2024). The study protocol for the entire VagLaser research program has been published elsewhere (reference omitted for peer review). The trial was approved by the Danish Data Protection Agency (1–16-02–327-22) and the Regional Committee on Health Research Ethics (1–10-72–183-22). The registered trial (NCT06007027) covers the broader VagLaser research program and includes multiple predefined sub-studies. This manuscript reports only the exploratory sub-study (vaginal punch biopsies) conducted within Study I (a prospective dose-response cohort) of the VagLaser research program, registered on 22 August 2023 at ClinicalTrials.gov (NCT06007027).

### Participants

Women with a history of primary breast cancer who were receiving adjuvant endocrine therapy (either selective oestrogen receptor modulators or aromatase inhibitors) and presenting with GSM symptoms were eligible for inclusion. Exclusion criteria were: pelvic organ prolapse (stage II or higher), vaginal oestrogen use within the past 12 months, chemotherapy within the past 6 months, active genital or urinary tract infections, or a history of vaginal reconstructive surgery. All participants provided written informed consent prior to study enrolment.

### Treatment intervention

Each participant received five sessions of fractional CO₂ laser therapy (SmartXIDE^2^V^2^LR,

MonaLisa Touch, DEKA, Florence, Italy) at 4–6 week intervals. Laser treatment was performed with the following settings: 30 W power, dwell time 1000 µs, and dot spacing 1000 μm. SmartStack 2 was applied for the first three sessions (fluence 4.41 J/cm^2^), and SmartStack 3 for the fourth and fifth (fluence 6.61 J/cm^2^). At the level of the vaginal introitus, dot power was reduced to 20 W. Neither analgesia nor vaginal gel was used. No pre-treatment regimen was applied, and participants did not use any non-hormonal lubricants, moisturisers, probiotics, or other local therapies during the study period. All participants applied the same intimate emollient cream (92% fat content; *Dr. Warming Critical Care*, Denmark) for comfort after biopsy and laser sessions.

### Tissue collection and Preparation

A 4-mm punch biopsy was obtained from the left lateral vaginal wall at baseline (four to six weeks before the first laser treatment) and from the right lateral vaginal wall at follow-up (four to six weeks after the fifth laser treatment). A local anaesthetic gel was applied before each biopsy. Biopsies were formalin-fixed and paraffin-embedded (Tissue-Tek Auto-TEC a120, Sakura Finetek USA, Inc., CA, USA). Sections of 2.5 μm were cut and stained with haematoxylin and eosin (HE, Dako CoverStainer, Agilent Technologies, Inc. CA, US), Masson’s trichrome (Ventana BenchMark Special Stains, F. Hoffmann-La Roche Ltd, AZ, US) and Verhoeff-Van Gieson elastin (Ventana BenchMark Special Stains). All staining procedures followed the manufacturer’s instructions.

Additional sections were immunohistochemically stained on the Ventana Benchmark Ultra Platform for collagen I (Col1A1, EPR7785, 1:300, Abcam Ltd, UK) and collagen III (Col3A1, IE7-D7/col3, 1:75, Abcam Ltd).

Sections for collagen I and collagen III were pretreated using HIER CC1 (pH 8.5 and pH 6.0, respectively) for 32 min at 97º C and 91º C, followed by antibody incubation for 32 min. Optiview DAB (Ventana) was used for visualisation, and slides were counterstained with haematoxylin II and a bluing reagent for both immunostains.

### Analogue microscopy

**Fig. 1:**
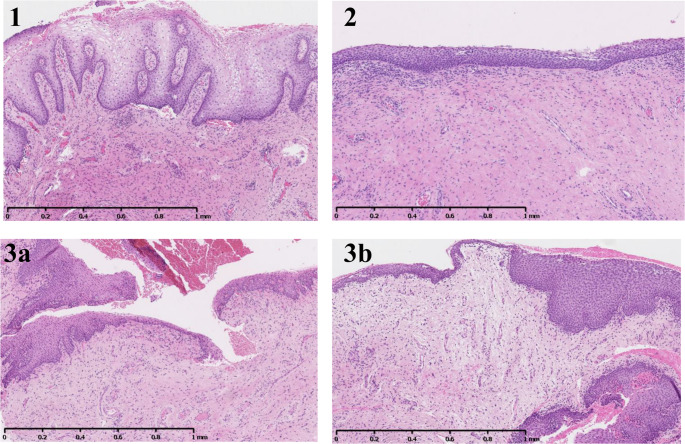
Histologic categories. **Representative histological images of vaginal mucosa from study participants. 1**: Type 1 well-glycogenated epithelium with rete ridges. **2**: Type 2 thinned squamous epithelium. **3a**: Type 3 transition from epithelium with rete ridges and glycogenisation to thinned epithelium. **3b**: Type 3 vaginal mucosa with thinned epithelium with focal cytoplasmic glycogen. All sections stained with HE; magnification x5

Two gynaecopathologists independently classified HE slides while blinded to time point and clinical data, using three epithelial morphology types described by Li et al. (Type 1–3) [13]. Discrepancies were resolved by joint consensus review. According to Li et al., Type 1 is characterised by normal squamous epithelium with abundant glycogen and well-vascularised stroma. Type 2 is characterised by thinned squamous epithelium with increased basal layer thickness. Type 3 is characterised by a combination of features of Type 1 and 2 (Fig. 1).

The vaginal epithelium was assessed for the following morphological features: glycogen load (Type 1: rich; Type 2: poor), presence of rete ridges and subepithelial papillae (Type 1: present; Type 2: absent), parakeratosis (Type 1: present; Type 2: absent not), keratinisation (Type 1: present; Type 2: absent), and basal layer thickness (Type 1: thin; Type 2: thick). In addition, the lamina propria was evaluated for vascularisation (Type 1: evident; Type 2: not evident).

Orientation note: Despite standardised sampling (lateral wall, 3 cm from the introitus), variation in section orientation and tangential cuts precluded reliable quantitative epithelial morphometry. Thus, epithelial thickness was not analysed.

### Whole-slide imaging

Nanozoomer 2.0HT (Hamamatsu Photonics KK, Hamamatsu City, Japan) generated whole-slide images of the two special stains (Masson’s trichrome and Verhoeff-Van Gieson) and of the immunohistochemical staining for collagen I and III at a magnification of 20X.

### Digital image analysis

Area-based elastin and collagen indices were automatically quantified using Visiopharm (version 2023.09.5.15777; Visiopharm A/S, Hørsholm, Denmark), including the AI Architect module. All analyses were performed blinded to the sample time point and clinical data.

Outline of the region of interest (vaginal stroma without large blood vessels, aggregates of red blood cells, and preanalytical and analytical artefacts) and the quantification of elastin (Verhoeff-Van Gieson) were based on convolutional neural networks [14], trained with manually generated pixel-level annotations. Loss-weighting and data augmentation were utilized. All feature maps of neural networks with added mean filters were subsequently classified by thresholding, and postprocessing further enhanced results (morphological operations and changes by area or surrounding).

Collagen was quantified by thresholding the blue chromaticity level in Masson’s trichrome, and by thresholding of a DAB deconvolution, the red colour level, and the contrast between red and blue colour levels, including post-processing (morphological operations and changes by area) in immunohistochemical stains.

Area-based indices were calculated by dividing the area of either elastin or collagen by the outlined stromal area. All analyses were performed blinded to the sample time point and clinical data.

### Statistical analyses

Categorical variables were presented as frequencies (n) and percentages (%), and continuous variables as means with 95% confidence intervals (95% CIs). Changes in histological classification (Type 1, Type 2, and Type 3) between baseline and follow-up were analysed using the Stuart–Maxwell test. Individual-level transitions in histological types were illustrated using a Sankey diagram. Normality of continuous data was assessed visually (Q–Q plots) and statistically (Shapiro–Wilk test). Changes in stromal tissue parameters – including elastin, total collagen, collagen type I and collagen type III indices – were evaluated using paired Student’s *t*-test (for normally distributed data) or the Wilcoxon signed-rank test (for non-normally distributed data). All statistical tests were exploratory and not adjusted for multiple comparisons, as the study was not powered for confirmatory inference. All analyses were performed using Stata version 18.0 (StataCorp LLC, College Station, TX, USA). A two-sided *p* < 0.05 was considered statistically significant.

## Results

A total of 26 breast cancer survivors completed all five sessions of fractional CO_2_ laser treatment and provided paired baseline and follow-up biopsies. Four participants discontinued before follow-up sampling and were excluded from the histological analyses.

Baseline characteristics are presented in Table 1.

**Table 1:** Baseline characteristics, *n* = 26

Age, y, mean, (SD) (range) At enrolment At menopause	54.5 (7.8) (40–72) 47.5. (4.3) (38–57)
*Mean BMI (kg/m*^2^ *) mean (SD)*	28 (4.7)
*Type of menopause*,* n (%)* * Natural* * Induced by the cancer treatment*	15 (57.7) 11 (42.3)
*Smoking status*,* n (%)* *Current smokers* *Former smokers* *Non-smokers*	3 (11.5) 13 (50) 10 (38.5)
*Time since breast cancer diagnosis*,* y*,* mean (SD) (range)*	3.4 (2.2) (1–10)
*Type of hormone therapy*,* n (%)* * Selective Estrogen Receptor Modulator* * Aromatase Inhibitor*	9 (34.6) 17 (65.4)

### Epithelial morphology

**Fig. 2:**
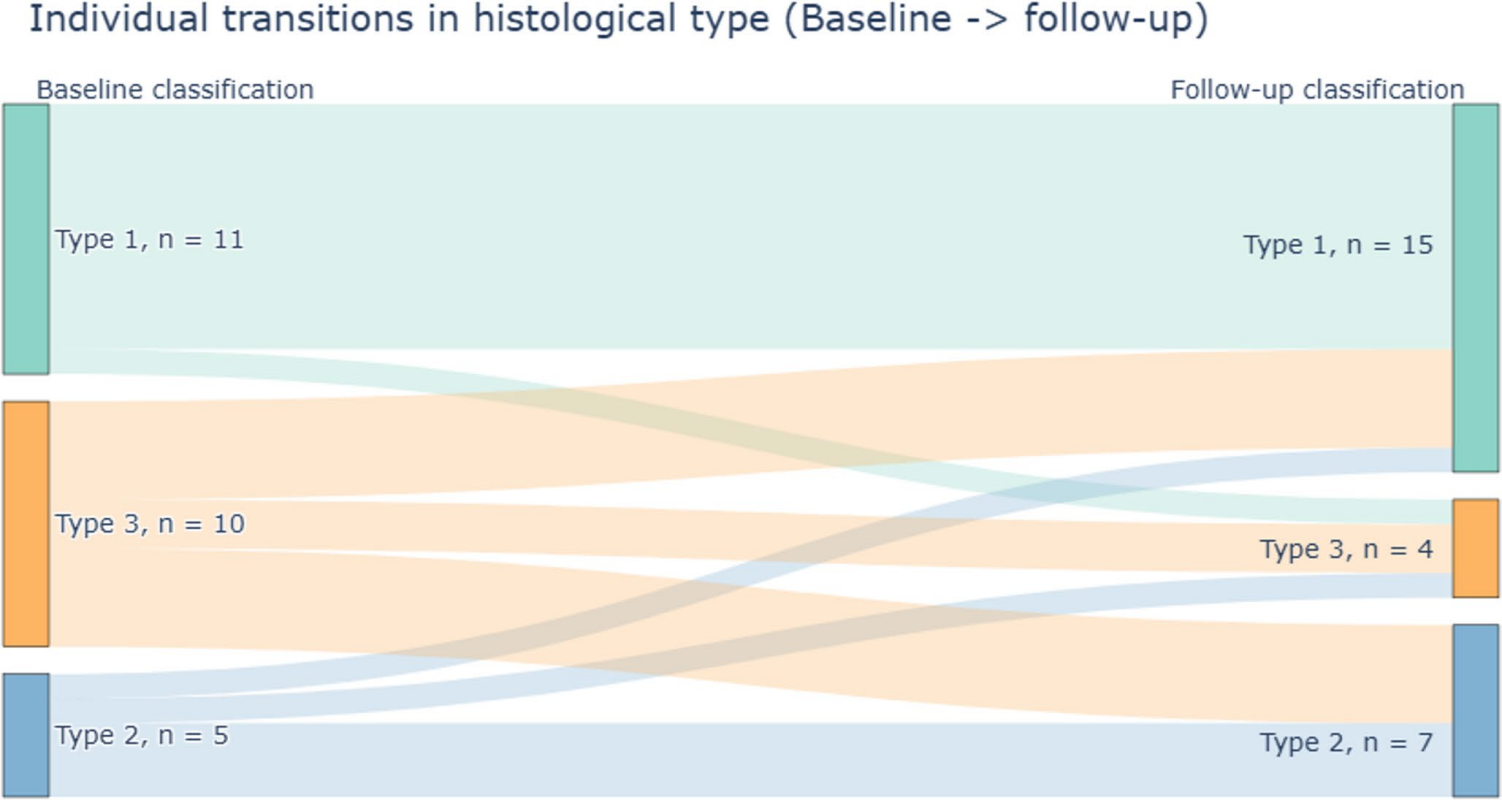
Sankey diagram visualising transitions in histological classification of the vaginal epithelium from baseline to follow-up biopsy after vaginal laser treatment. Type 1 (green), Type 2 (blue), and Type 3 (yellow) as defined in Fig. 1

The two pathologists agreed on the classification of 43 of the 52 biopsies (83%) and consensus was required for the remaining nine. At baseline 42.3% of participants (*n* = 11) were classified as Type 1, 19.2% (*n* = 5) as Type 2, and 38.5% (*n* = 10) as Type 3 (Fig. 1). After treatment, the distribution of epithelial types did not change significantly (Stuart–Maxwell test *p* = 0.16). Individual transitions between histological types over time were illustrated using a Sankey diagram, showing participant flow between Type 1, Type 2, and Type 3 from baseline to follow-up, including both stable and changing patterns (Fig. 2).

Quantitative assessment of epithelial thickness was not feasible due to variable biopsy orientation and occasional tangential sectioning. Therefore, epithelial morphology was evaluated only by analogue classification.

### Stromal morphology

**Fig. 3:**
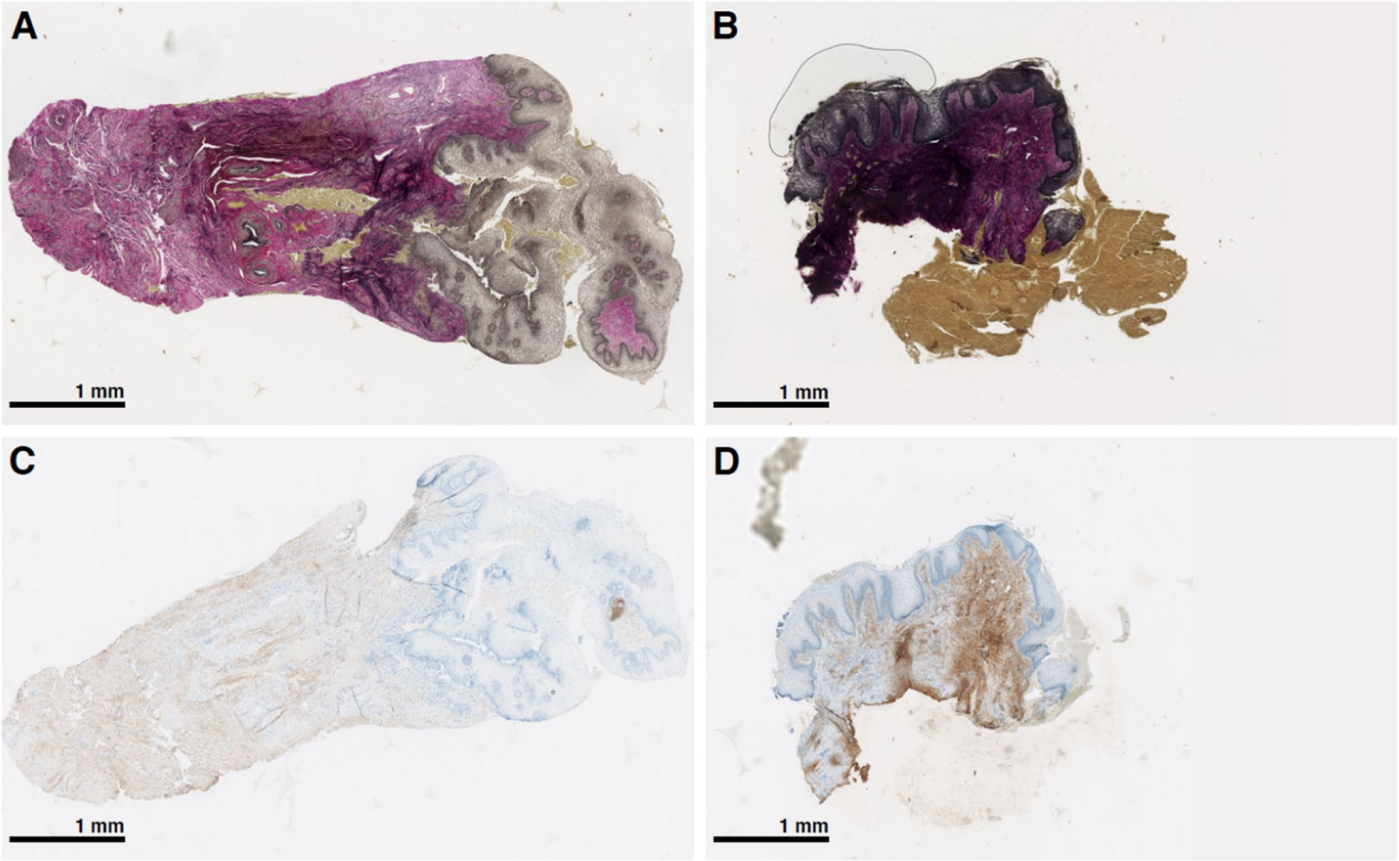
Elastin and collagen type III from baseline to follow-up. **Representative histological images of vaginal stroma tissue from one of the study participants.** **A**: Elastin at baseline. **B**: Elastin at follow-up **C**: Collagen type III at baseline. **D**: Collagen type III at follow-up. A-B sections stained with Verhoeff-Van Gieson, C-D sections immunohistochemically stained. Magnification x5

Digital image analysis demonstrated significant increases in stromal elastin and collagen type III indices from baseline to follow-up (Fig. 3), whereas total collagen and collagen type I indices showed no significant change. The elastin index increased from 33.1% at baseline to 56.8% at follow-up (*p* < 0.01). The collagen type III index increased from 44.5% to 58.4% (*p* = 0.03) (Table 2).

**Table 2:** Elastin and collagen-related parameters at baseline and follow-up

	Baseline, mean (95% CI)	Follow-up, mean (95% CI)	Mean difference (95% CI)	*p*-value
Elastin index (%)	33.1 (25.8; 40.5)	56.8 (46.9; 66.6)	23.7 (11.1; 36.2)	**< 0.01**
Total Collagen index (%)	63.4 (57.2; 69.6)	54.5 (47.7; 61.3)	−8.9 (−18.8; 0.9)	0.07
Collagen type I index (%)	50.3 (39.2; 61.3)	54.6 (44.8; 64.3)	4.3 (−4.6; 13.2)	0.33
Collagen type III index (%)	44.5 (34.9; 54.1)	58.4 (51.1; 65.6)	13.8 (1.7; 26)	**0.03**

Values are presented as means with 95% confidence intervals (CIs). Differences between baseline and follow-up were analysed using paired t-tests

## Discussion

This exploratory histological study investigated how fractional vaginal CO₂ laser therapy affects the vaginal wall of BCSs with GSM. The main finding was a significant increase in stromal elastin and collagen type III content, whereas epithelial morphology remained unchanged. These results indicate a selective remodelling of stromal morphology following treatment, without clear evidence of epithelial regeneration.

Our results are consistent with those of Bretas et al. [2], who reported a significant increase in collagen type III fibers after three sessions of fractional CO₂ vaginal laser treatment, without a corresponding change in collagen type I. They also observed a decreased collagen I: III ratio, suggesting a shift toward a more flexible connective tissue composition. Similarly, Benitez-Roig et al. [7] found enhanced collagen and elastin content in the lamina propria, together with increased epithelial thickness, after two sessions of CO₂ vaginal laser in postmenopausal women with GSM.

Notably, these studies investigated postmenopausal women, whereas our cohort comprised BCSs who, in addition to their postmenopausal status, were receiving anti-oestrogen therapy. Despite this difference, the consistent finding of increased collagen type III and elastin content across studies suggests that CO₂ laser therapy may induce comparable stromal remodeling in different hypo-estrogenic populations. Enhanced elastin and collagen III content, in particular, may contribute to improved tissue resilience and biomechanical support of the vaginal wall, even in the profoundly oestrogen-deprived environment typical of BCSs [15].

Only two previous studies in BCSs have evaluated histological changes following CO₂ laser therapy. Mension et al. [11] assessed elasticity and found no significant differences between active and sham treatment, and Cantarelli et al. [12] reported no post-treatment stromal changes between the three groups. These outcomes are not directly comparable with our study, as we focused on indices of elastin and collagen subdivided by type I and III. Moreover, whereas Mension et al. and Cantarelli et al. both assessed histology using HE staining, we supplemented HE with Verhoeff-Van Gieson for quantitative elastin and Masson’s trichrome plus immunohistochemistry for collagen, enabling a more detailed evaluation of extracellular matrix composition through digital image analysis.

In the study by Li et al. [13], a poor correlation was found between histological features of the vaginal epithelium and the severity of subjective GSM symptoms in postmenopausal women. Notably, 27% of their participants exhibited a well-estrogenised Type 1 despite reporting significant vaginal symptoms. In our cohort, this proportion was even higher, with 42.3% of participants classified as Type 1 at baseline. Similarly, Cantarelli et al. [12] observed that most pre-treatment vaginal biopsies (> 90%) from BCSs with GSM did not show histological atrophy. This discrepancy underscores the limitations of the Li et al. classification system, which may not fully capture the biological heterogeneity or symptom-related pathology of the postmenopausal or hormonally suppressed vaginal mucosa. It also suggests that GSM in BCSs may arise from multifactorial or poorly understood mechanisms, beyond epithelial thinning alone.

Two factors constrain the interpretation of epithelial outcomes in this study. First, inconsistent biopsy orientation and occasional tangential sectioning prevented quantitative assessment of epithelial thickness. Second, without a sham-treated control group, it is not possible to differentiate between laser-specific effects and nonspecific tissue-healing effects. The short follow-up interval (1–2 months) further limits insight into the persistence or functional relevance of the observed stromal changes. Finally, the relatively small sample size (*n* = 26) reduces statistical power and generalisability. Together, these constraints mean that the current results should be regarded as preliminary histological evidence of stromal remodelling rather than proof of therapeutic regeneration.

This study also has several strengths. All staining, imaging, and quantification were performed under blinded conditions using validated digital algorithms, ensuring reproducibility. Participants represented a clinically relevant population of BCSs on anti-oestrogen therapy, in whom hormonal treatments are contraindicated. The protocol was prospectively registered, and tissue sampling was standardised to minimize analytical variability.

In summary, fractional CO_2_ vaginal laser therapy in BCSs induced measurable alterations in stromal morphology, characterized by increased elastin and collagen III indices, while epithelial morphology remained unchanged. These findings suggest a selective stromal response detectable by quantitative histology. Future controlled trials combining structural, microbiota, and clinical endpoints are needed to establish the biological and functional relevance of these stromal changes.

## Conclusion

Fractional CO_2_ vaginal laser therapy in BCSs produced a stromal response, with increased elastin and collagen III content and no epithelial changes. These results provide preliminary histological evidence of tissue-level remodelling and warrant confirmation in larger controlled studies.

## Supplementary Information

Below is the link to the electronic supplementary material.

No gels or blots were generated or analysed in this study; therefore, provision of uncropped images is not applicable.

ESM 1 (JPG 656 KB)

ESM 2 (PJG 0.98 MB)

ESM 3 (PJG 774 KB)

ESM 4 (PJG 1.31 MB)

## Data Availability

Data are under patient protection. Can be provided upon request.

## Declarations

### **Clinical trial registration**

ClinicalTrials.gov Identifier: NCT06007027. Registered on 22 August 2023.
