# Supplementary figures and images for: Impact of fractional CO2 laser therapy on vaginal wall histology in breast cancer survivors
Source: Lasers Med Sci. 2026 Feb 10;41(1):26. doi: 10.1007/s10103-026-04829-0 (PMC12890975; doi:10.1007/s10103-026-04829-0)

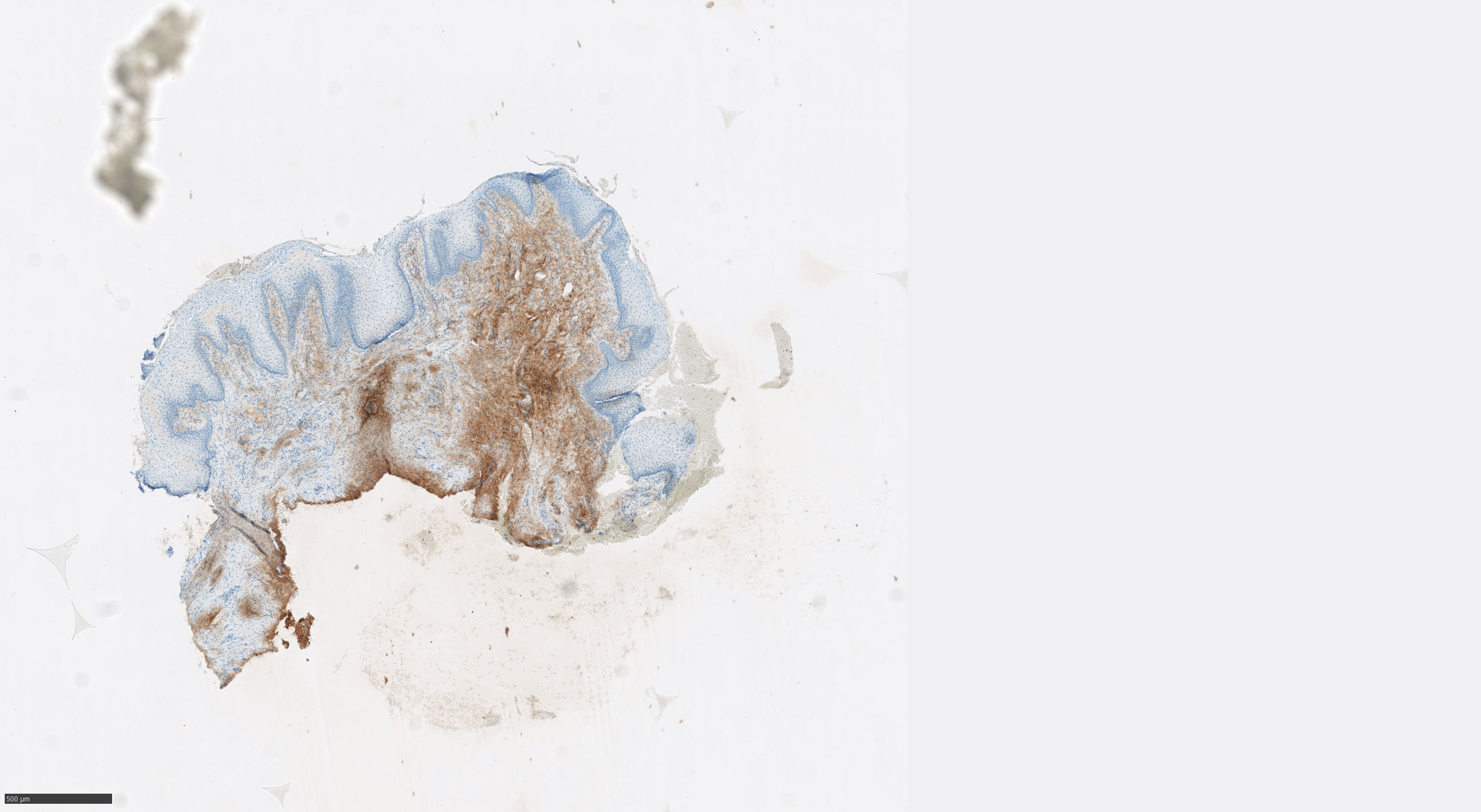

Supplement: Supplementary file 1 — (JPG 656 KB) [file 10103_2026_4829_MOESM1_ESM.jpg]

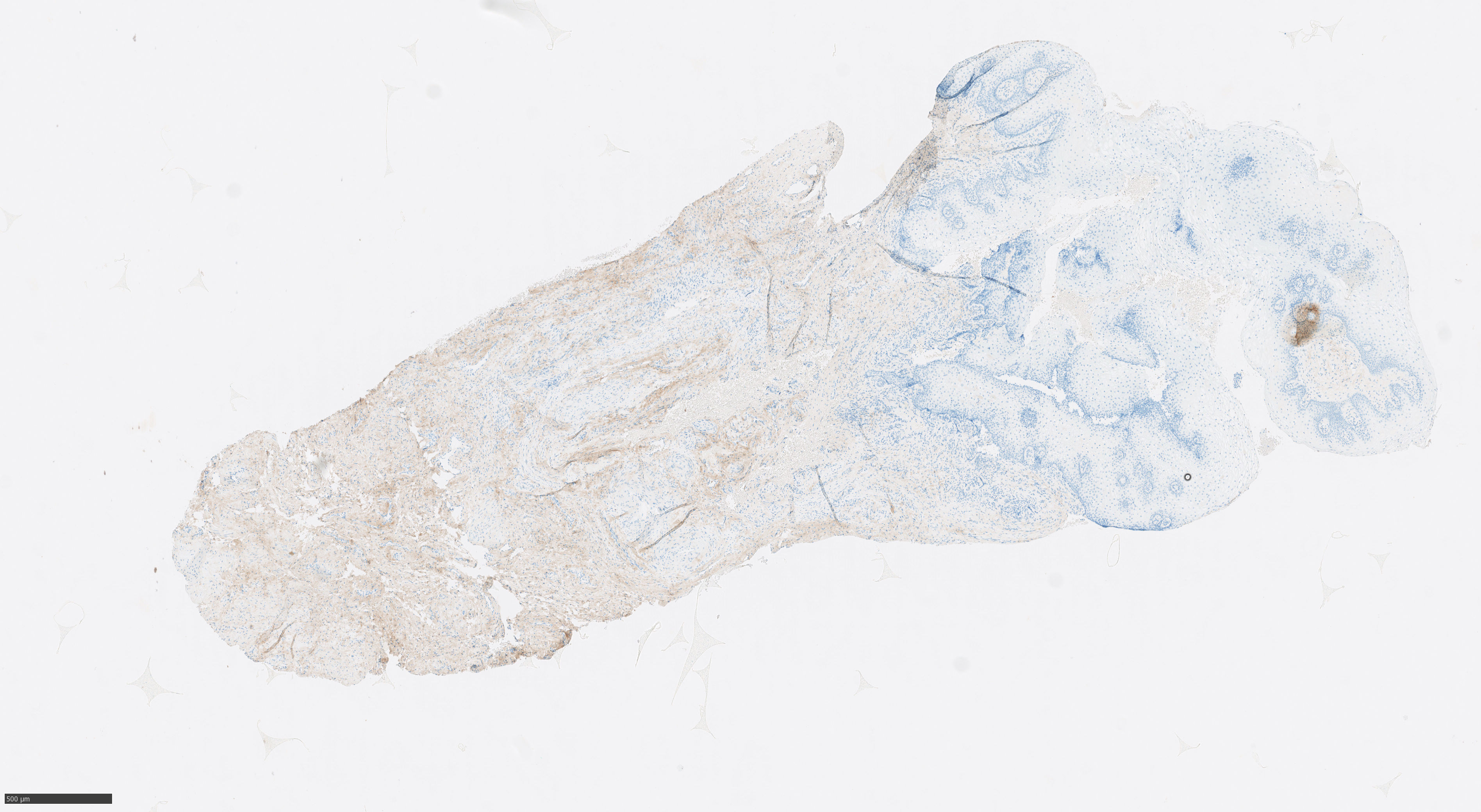

Supplement: Supplementary file 2 — (PJG 0.98 MB) [file 10103_2026_4829_MOESM2_ESM.jpg]

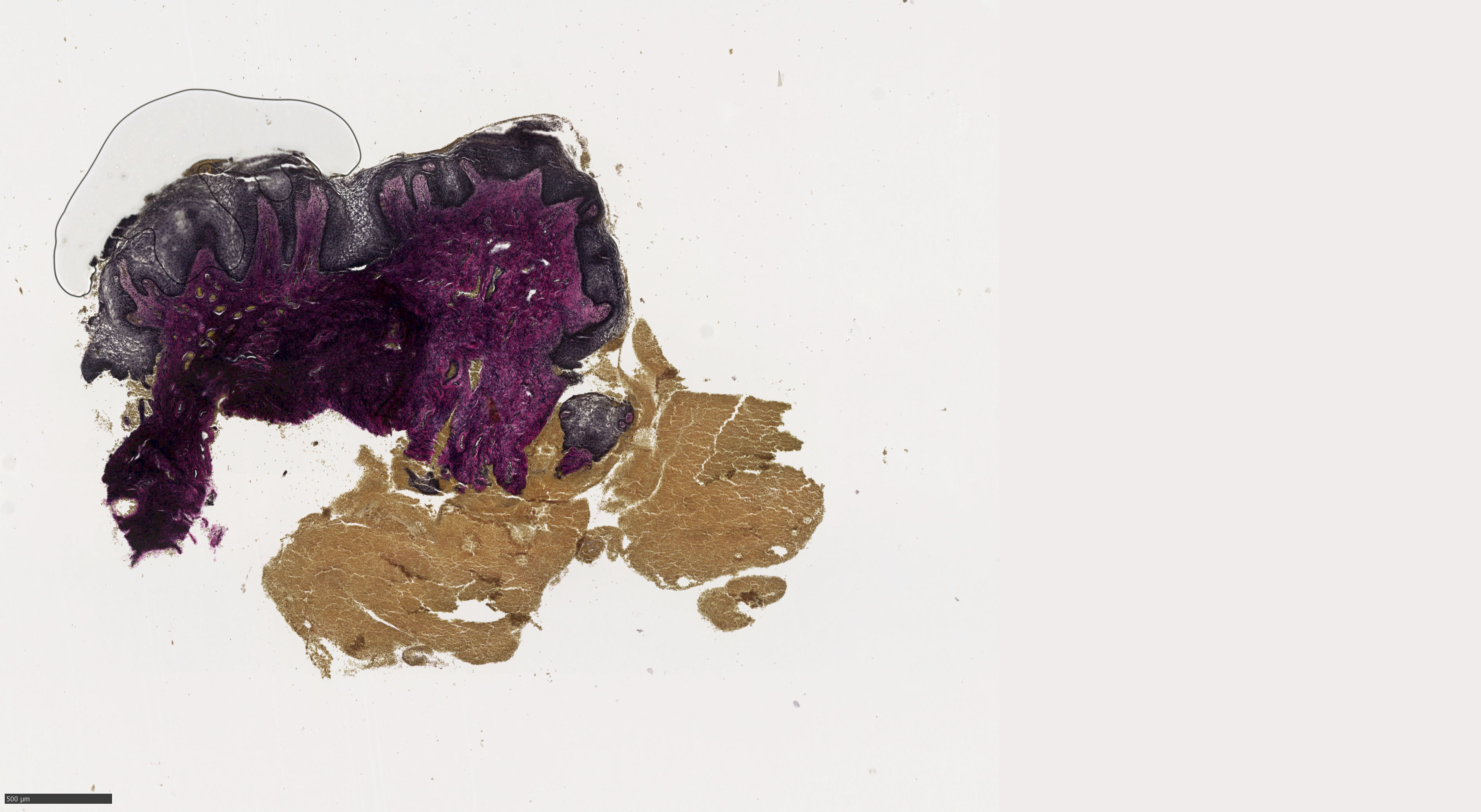

Supplement: Supplementary file 3 — (PJG 774 KB) [file 10103_2026_4829_MOESM3_ESM.jpg]

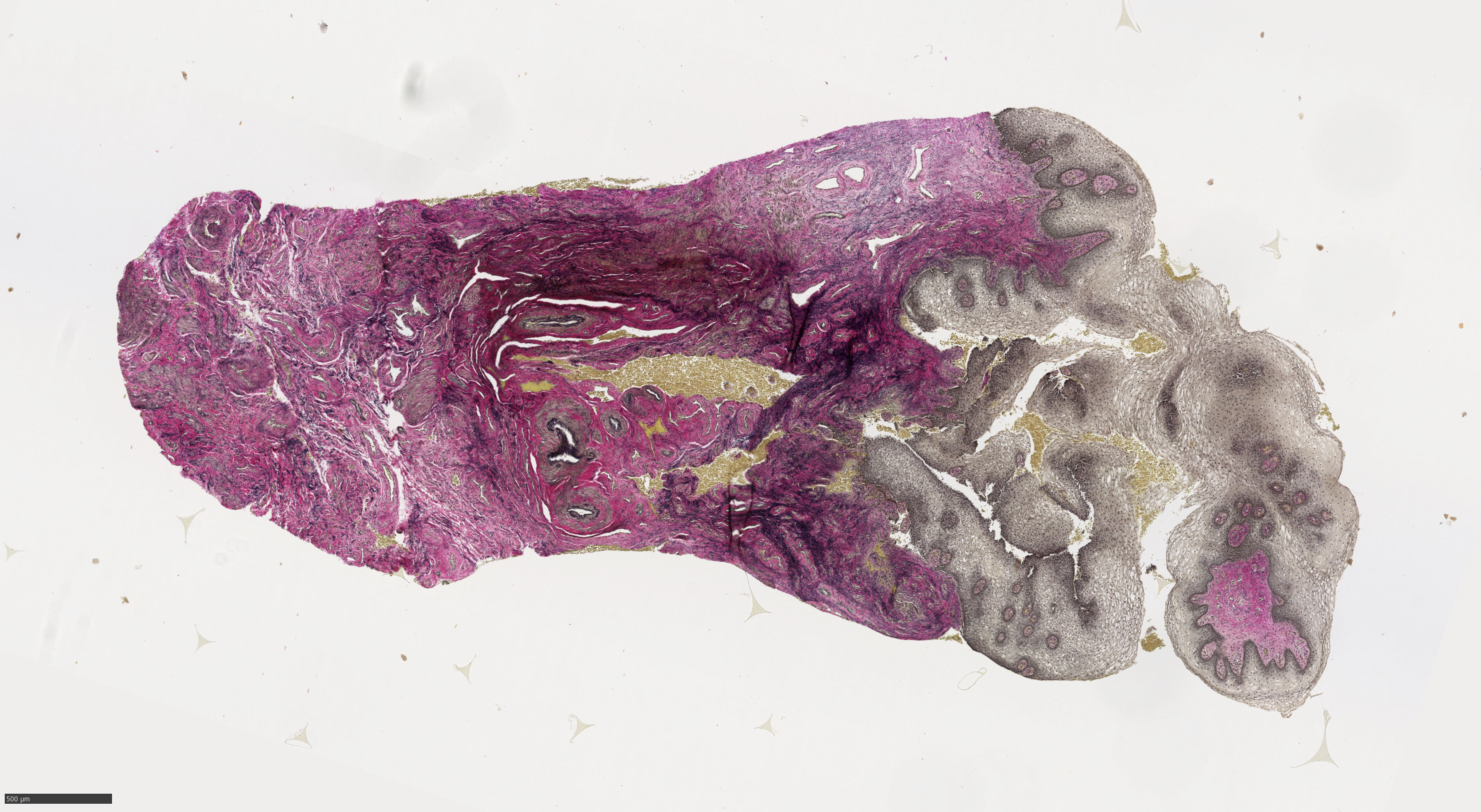

Supplement: Supplementary file 4 — (PJG 1.31 MB) [file 10103_2026_4829_MOESM4_ESM.jpg]
